# Class II histone deacetylases require P/Q-type Ca^2+^ channels and CaMKII to maintain gamma oscillations in the pedunculopontine nucleus

**DOI:** 10.1038/s41598-018-31584-2

**Published:** 2018-09-03

**Authors:** Francisco J. Urbano, Verónica Bisagno, Susan Mahaffey, Sang-hun Lee, Edgar Garcia-Rill

**Affiliations:** 1 Center for Translational Neuroscience, Department Neurobiology & Dev. Sci., University of Arkansas for Medical Sciences, Little Rock, AR USA; 2 IFIBYNE, CONICET, Universidad de Buenos Aires, Buenos Aires, Argentina; 3 ININFA, CONICET, Universidad de Buenos Aires, Buenos Aires, Argentina; 4 Department Neurology, University of Arkansas for Medical Sciences, Little Rock, AR USA

## Abstract

Epigenetic mechanisms (i.e., histone post-translational modification and DNA methylation) play a role in regulation of gene expression. The pedunculopontine nucleus (PPN), part of the reticular activating system, manifests intrinsic gamma oscillations generated by voltage-dependent, high threshold N- and P/Q-type Ca^2+^ channels. We studied whether PPN intrinsic gamma oscillations are affected by inhibition of histone deacetylation. We showed that, a) acute *in vitro* exposure to the histone deacetylation Class I and II inhibitor trichostatin A (TSA, 1 μM) eliminated oscillations in the gamma range, but not lower frequencies, b) pre-incubation with TSA (1 μM, 90–120 min) also decreased gamma oscillations, c) Ca^2+^ currents (I_Ca_) were reduced by TSA, especially on cells with P/Q-type channels, d) a HDAC Class I inhibitor MS275 (500 nM), and a Class IIb inhibitor Tubastatin A (150–500 nM), failed to affect gamma oscillations, e) MC1568, a HDAC Class IIa inhibitor (1 μM), blocked gamma oscillations, and f) the effects of both TSA and MC1568 were blunted by blockade of CaMKII with KN-93 (1 μM). These results suggest a cell type specific effect on gamma oscillations when histone deacetylation is blocked, suggesting that gamma oscillations through P/Q-type channels modulated by CaMKII may be linked to processes related to gene transcription.

## Introduction

Epigenetic mechanisms (i.e., histone post-translational modification and DNA methylation) play a role in regulation of gene expression in response to a wide range of environmental stimuli, such as learning, stress, or drugs of abuse^1^. In particular, acetylation/deacetylation of histones is associated with the modulation of transcription in multiple ways, by relaxing chromatin structure that would increase the accessibility of transcription factors to their target genes, and also by direct acetylation of transcription factors^2^. Histone acetylation/deacetylation is a dynamic process controlled by the antagonistic actions of two large families of enzymes: the histone acetyltransferases (HATs) and the histone deacetylases (HDACs). The balance between the actions of these enzymes serves as a key regulatory mechanism for many cellular processes and disease states. The HDACs have been classified into four classes (Class I-IV), based on localization and amino acid sequence similarities^2,3^. While Class I HDACs (HDACI) are localized in the nucleus, Class IIs (HDACII) shuttle between the nucleus and the cytoplasm where they can modify non-histone proteins.

HDACI/II knockdown using lentiviral transfection of cultured hippocampal neurons altered excitatory synaptic transmission and the formation of dendritic spines^4^. These authors showed that HDACI/II had different synaptic roles during early developmental stages compared to what was observed in neuronal cultures kept *in vitro* over 16 days. Synaptic effects of Class III (HDACIII) knockdown were mimicked by 18–24 hour pre-incubation of cultures with the histone deacetylation inhibitor trichostatin A (TSA). Other authors have failed to observe similar results after chronic treatment with TSA of hippocampal organotypic cultures, suggesting the existence of molecular compensations associated to the permanent knockdown of HDACs^5^. Knockdown of many of the individual HDAC isoforms are lethal, emphasizing the importance of performing acute experiments using HDAC inhibitors in order to assess their role in the modulation intrinsic neuronal properties.

The epigenetic profile of numerous HDAC inhibitors is being explored for translational applications^6^. HDACs and their inhibitors are promising candidates in treating cancer and several neurodegenerative disorders^7^. Many of these inhibitors have proven to have synergistic effects with other drugs^8^. However, the mechanisms of action of HDAC inhibitors need to be elucidated. Indeed, there are potential neurological side effects by modulating HDAC functions, and more research is needed to design more specific HDAC inhibitors with the lowest range of neurological side effects^7^. The neurological sequelae induced by HDAC inhibitors include fatigue, status epilepticus, somnolence, and gait problems^8^. That is, some side effects interfere with arousal and might be linked to altered PPN physiology since this nucleus modulates states of arousal.

The present study was designed to determine if modulation of HDACs affects the manifestation of gamma band oscillations in PPN neurons. The PPN is the only reticular activating system (RAS) nucleus that is active during high frequency (gamma range) EEG activity in both waking and rapid eye movement (REM) sleep^9,10^. The locus coeruleus and raphe nuclei are active during waking and slow wave sleep, but not during REM sleep. The PPN is the only cell group in the RAS in which every neuron manifests intrinsic gamma oscillations^9,10^. These oscillations are mediated only by voltage-dependent, high threshold P/Q-type and N-type Ca^2+^ channels^9–12^. We addressed the question; does blockade of HDACs affect the manifestation of gamma oscillations generated by one or both of these types of channels? If so, what are the intracellular signaling pathways involved in these effects?

## Results

Recordings of gamma band oscillations in PPN neurons (total number of cells studied n = 150) were performed using PPN slices at 36 °C in the presence of synaptic blockers (SB, namely, the selective NMDA receptor antagonist 2-amino-5-phosphonovaleric acid (APV, 40 μM), the competitive AMPA/kainate glutamate receptor antagonist 6-cyano-7-nitroquinoxaline-2,3-dione (CNQX, 10 μM), the glycine receptor antagonist strychnine (STR, 10 μM), the specific GABAA receptor antagonist gabazine (GBZ, 10 μM), and the nicotinic receptor antagonist mecamylamine (MEC, 10 μM) and the sodium channel blocker tetrodotoxin (TTX). Whole cell current clamp recordings of PPN cells showed that no significant rundown of oscillatory activity PPN neurons was evident during the recording period. Only one neuron per slice was used during acute application of HDAC inhibitors in order to prevent uncontrolled effects by their long lasting pre-incubation. Recordings from cells after pre-incubation were compared to those acquired in control slices. Please see the Methods section for the choice of concentration of each agent based on affinity and IC_50_.

### Inhibitory effects on intrinsic gamma oscillations by acute and prolonged application of HDAC Class I and II inhibitors

**Figure 1:**
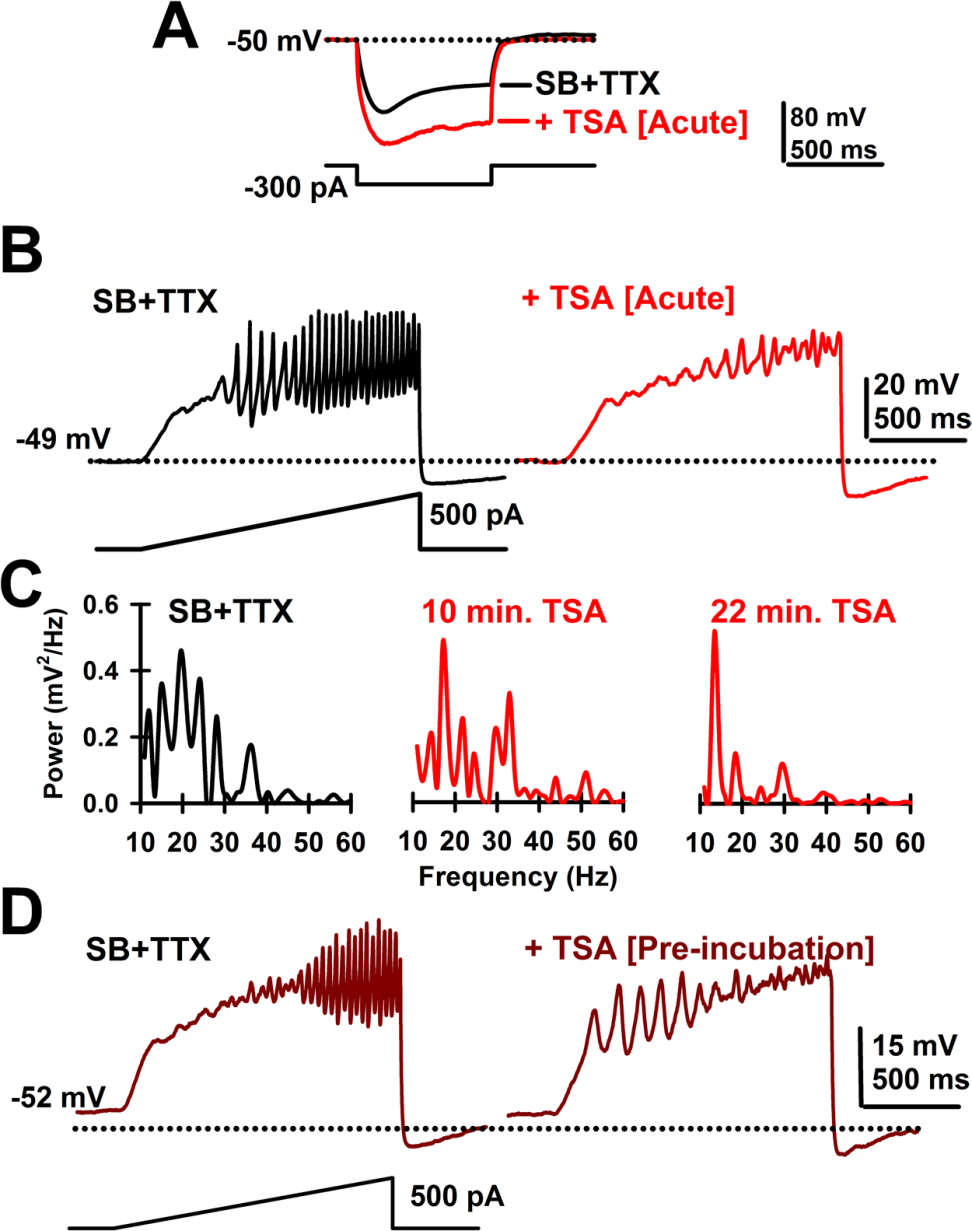
Effects of acute TSA. (**A**) Voltage records in response to hyperpolarizing steps after SB + TTX (black record) and following TSA (20 min; 1 μM, red record) showing the increase in resistance induced by acute TSA exposure. (**B**) Ramp-induced oscillations after SB + TTX (black record) as previously reported for PPN neurons and effects of acute TSA exposure (20 min; 1 μM, red record) showing elimination of fast oscillations and decreased amplitude of oscillations. (**C**) Representative plots of power spectrum over time for acute TSA (1 μM) bath application. (**D**) Ramp-induced oscillations in a representative PPN cell showing low amplitude, low frequency oscillations in the presence of SB + TTX after prolonged exposure to TSA (120 min; left side record).

We started by testing a widely used inhibitor of HDAC Class I and II, trichostatin A (TSA)^4,5^. No membrane potential changes were observed after acute TSA (1 μM) application (n = 8 PPN cells, SB + TTX: −51.9 ± 1.3 mV; +TSA: −50.3 ± 0.9 mV; paired Student’s t-test, df = 7; t = −1.52; p = 0.172). Bath application of TSA significantly increased the input resistance of PPN neurons (Fig. 1A), by 63 ± 23% (n = 7 PPN cells, paired Student’s t-test, df = 6; t = −3.19; p = 0.019), while blocking PPN membrane oscillations (Fig. 1B), reaching its maximum blocking effect after 20 min (Fig. 1C). No statistically significant decreases were observed in the power of lower frequency (10–15 Hz, 15–20 Hz, or 20–30 Hz) membrane oscillations (Table 1). However, the reduction in the gamma band range (30–100 Hz) was statistically significant (Table 1). Lower concentrations of TSA (250 nM) did not affect gamma band power (Paired Student’s t-test, n = 5; df = 4; t = 1.03; p = 0.36).

**Table 1:** Mean power changes (% of control) mediated by Trichostatin-A (TSA, 1 μM).

	10–15 Hz	15–20 Hz	20–30 Hz	30–100 Hz
Acute effect (n = 7)	76.6 ± 19.9%	53.2 ± 20.1%	49.7 ± 14.7%	36.4 ± 14.5%^#^
Pre-incubation effect (n = 19)	99.0 ± 11.3%	75.7 ± 23.2%	95.9 ± 12.6%	45 ± 3.4%*

^#^p < 0.05; paired Student’s *t*-test (df = 6, t = −3.53; p = 0.012). *p < 0.05; Student’s t-test (df = 18; t = −2.6, p = 0.02).

Class I and II HDACs have been described to act through their association with other proteins at nuclear and/or cytoplasmic sites^2^. Therefore, TSA might require longer periods of incubation prior to reaching its maximal effects. With this in mind, we pre-incubated PPN slices with 1 μM TSA for 90–120 min at 36 °C in the presence of SB + TTX. Membrane oscillations were recorded from PPN neurons (n = 8) in slices without pre-incubation and compared to those from slices following TSA pre-incubation, allowing us to compare changes in power spectrum with and without long-term TSA exposure. Figure 1D shows a representative PPN cell with no gamma band frequency oscillations after 120 min pre-incubation with TSA. On average, low frequency band power was unaffected, while only gamma band power was significantly reduced by TSA pre-incubation (Table 1). Neither membrane potential (without TSA: −53.1 ± 1.7 mV; n = 9; after TSA pre-incubation: −50.5 ± 1.2 mV; n = 12; One-way ANOVA; F(1, 19) = 1.6; p = 0.22), nor input resistance (without TSA: 135 ± 9 MΩ; n = 8; after TSA pre-incubation: 147 ± 8 MΩ; n = 12; One-way ANOVA; F(1, 18) = 0.8; p = 0.39), were altered by TSA pre-incubation.

### Effects of HDAC Class I and II inhibitors on PPN cells expressing different calcium channels

**Figure 2:**
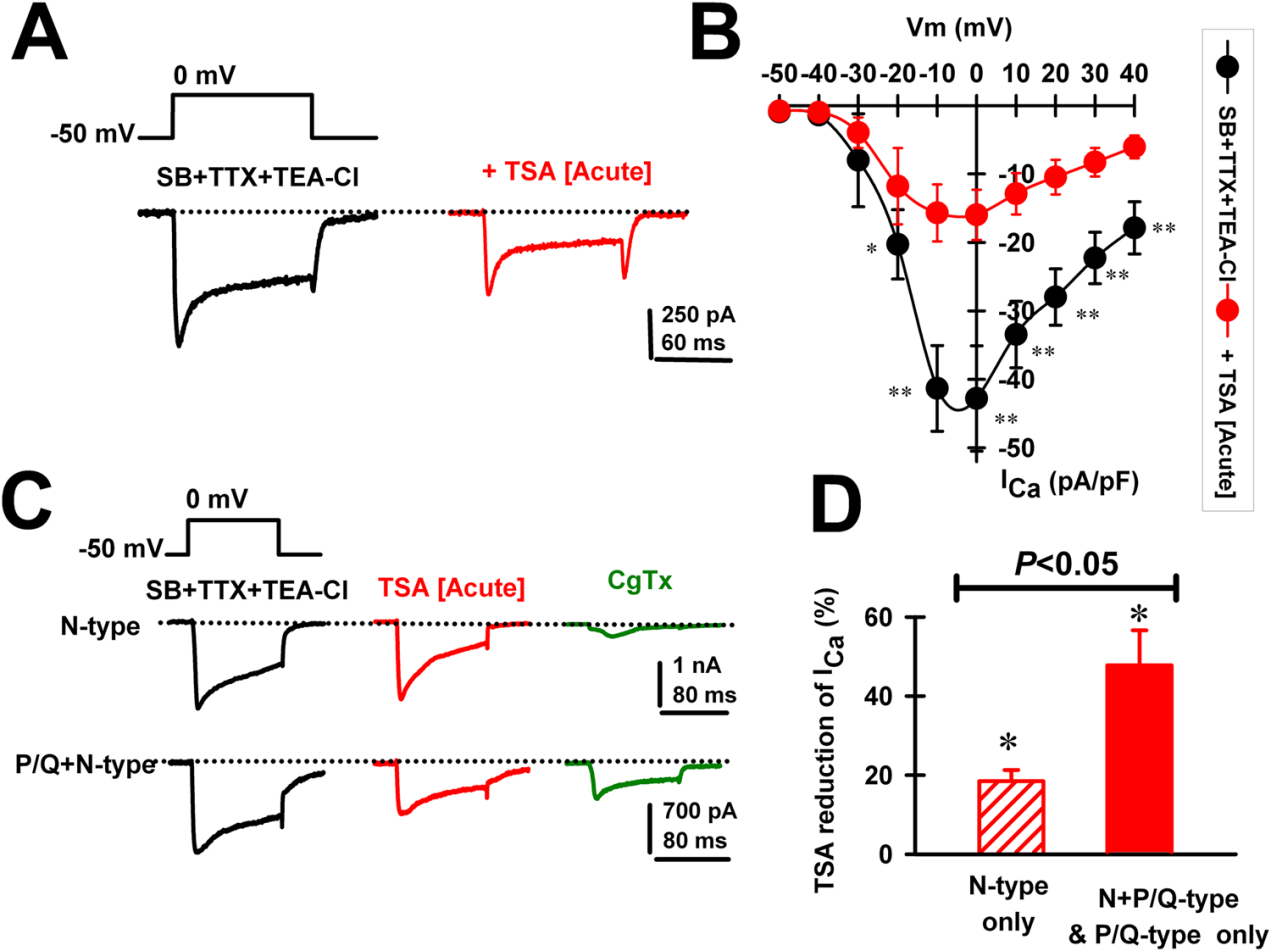
Effects of acute TSA (1 μM) on voltage-dependent Ca^2+^ currents (I_Ca_) in different PPN cell types. (**A**) Representative I_Ca_ recorded using a 100 msec long depolarizing square step from a holding potential of −50 mV to 0 mV before (black record) and after (red record) acute bath application of TSA (1 μM). (**B**) Standard current-voltage (I-V) relationship of I_Ca_ before (black circles; *n* = 8 PPN neurons), and after TSA (20 min; 1 μM, red circles, *n* = 8 PPN neurons). *p < 0.05, **p < 0.01 One-way ANOVA, Bonferroni post-hoc test comparing SB + TTX vs acute TSA (1 μM). Note significant decreases in I_Ca_ after TSA from −20 mV to 40 mV. (**C**) Representative I_Ca_ recorded from a N-only PPN and a N + P/Q PPN neuron using a 100 msec long depolarizing square steps from a holding potential of −50 mV to 0 mV in the presence of SB, TTX and TEA-Cl (black record), after acute bath application TSA (15 min., 1 μM; red record), and after ω-conotoxin GVIA (20 min., CgTx, 2.5 μM, green record) to block N-type channels. Note marked effect on the N-only cells, but partial effect on the N + P/Q cell. (**D**) Percent reduction in I_Ca_ amplitude by TSA (1 μM) in a group of PPN cells expressing only N-type (n = 7; dashed red bar), or P/Q-type (n = 10; solid red bar) Ca^2+^ channels. *p < 0.05 significant reduction of I_Ca_ by acute bath application of TSA for N-only PPN neurons (Paired Student’s t-test; n = 7; df = 6; t = −3.62; *p* = 0.015), or PPN cells expressing P/Q-type channels (i.e., P/Q-only plus N + P/Q types) (paired Student’s t-test; n = 10; df = 9; t = −2.74; *p* = 0.029). One-way ANOVA comparison of both groups showed significant differences (F(1,15) = 7.2; p < 0.05; Bonferroni’s post-hoc t-test, t = 2.68, p = 0.02).

We then tested whether the TSA-mediated reduction in gamma oscillations might be a consequence of its blocking effect on Ca^2+^ currents (I_Ca_) mediated by high-threshold, voltage-dependent channels. I_Ca_s were recorded after gaining access to the neuronal intracellular space and series resistance was compensated and stable. We used square voltage steps with high Cs^+^ intracellular pipette solution and synaptic receptor blockers (SB) plus TTX (see Methods). Recordings of I_Ca_ were carried out for up to 30 min without significant rundown, similarly to previously reports^13,14^. The holding potential was at first set at −50 mV and then depolarized using square pulses up to 40 mV. Bath application of TSA (1 μM) was found to reduce I_Ca_ (Fig. 2A), without affecting their kinetics (Table 2). In a group of PPN neurons (n = 8), the I_Ca_ blocking effects of TSA were observed throughout a wide range of test potentials (Fig. 2B). There were significant reductions in I_Ca_ between −20 mV and +40 mV. Importantly, recorded PPN cells expressing P/Q type Ca^2+^ channels showed greater TSA blocking effects on I_Ca_ compared to those expressing only N-type channels (Fig. 2C,D). N-type only PPN neurons manifested an 18.5 ± 2.8% TSA-mediated reduction of I_Ca_ (Paired Student’s t-test; n = 7; df = 6; t = −3.62; *p* = 0.015). PPN cells expressing P/Q-type channels (i.e., P/Q-only plus N + P/Q cell types) showed a 47.8 ± 8.8% reduction after acute TSA (1 μM) application (Paired Student’s t-test; n = 10; df = 9; t = −2.74; *p* = 0.029), a significantly greater effect (One-way ANOVA, F(1,15) = 7.2; p < 0.05; Bonferroni’s post-hoc t-test, t = 2.68, p = 0.02).

**Table 2:** Time course of activation (τ_ON_) and deactivation (τ_OFF_) of calcium currents before and after acute application of HDAC inhibitors.

	SB + TTX + TEA-Cl	+TSA (1 μM)	SB + TTX + TEA-Cl	+MC1568 (1 μM)	SB + TTX + TEA-Cl + KN93 (1 μM)	+TSA (1 μM)
τ_ON_ (ms)	3.2 ± 0.9 (n = 7)	8.7 ± 2.8 (n = 7; p > 0.05)	3.1 ± 1.4 (n = 8)	8.7 ± 3.4 (n = 8; p > 0.05)	2.8 ± 0.8 (n = 7)	3.6 ± 1.0 (n = 7; p > 0.05)
τ_OFF_ (ms)	2.2 ± 0.6 (n = 7)	5.1 ± 1.3 (n = 7; p > 0.05)	2.6 ± 0.9 (n = 8)	6.2 ± 1.7 (n = 8; p > 0.05)	1.1 ± 0.3 (n = 7)	1.7 ± 0.3 (n = 7; p > 0.05)

Paired Student’s t-test comparisons showed no significant differences:

Acute TSA (1 μM): τ_ON_; t = 2.3, df = 6, p = 0.06, τ_OFF_; t = 2.4, df = 6, p = 0.055.

Acute MC1568 (1 μM): τ_ON_; t = 2.2, df = 7, p = 0.06, τ_OFF_; t = 2.3, df = 7, p = 0.06.

Acute KN93 (1 μM) + TSA (1 μM): τ_ON_; t = 2.2, df = 6, p = 0.07, τ_OFF_; t = 1.3, df = 6, p = 0.23.

### Effects of specific HDAC Class I and Class II inhibitors

**Figure 3:**
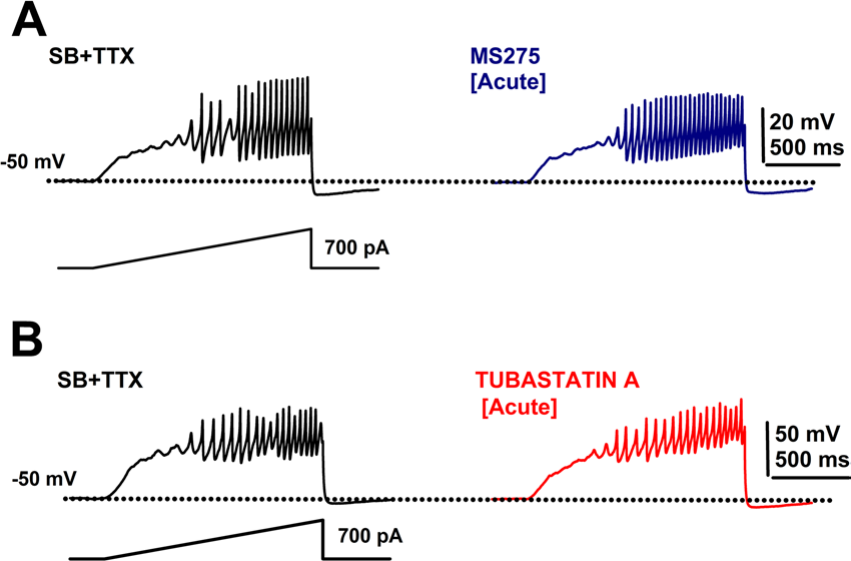
Effects of acute MS275 and tubastatin A. (**A**) Ramp-induced oscillations after SB + TTX (black record) as previously reported for PPN neurons, and effects of acute MS275 exposure (20 min; 500 nM, blue record) showing no effect on gamma oscillations. (**B**) Ramp-induced oscillations before (SB + TTX; black record) and after acute tubastatin A exposure (20 min; 150 nM, blue record) showing no effect on gamma oscillation amplitude or frequency.

We then tested a specific HDAC Class I inhibitor, MS275 (500 nM)^2^. Bath application of MS275 did not affect the input resistance of PPN neurons (n = 6 PPN cells, paired Student’s t-test, df = 5; t = −2.26; p = 0.074). No effect on PPN membrane oscillations was observed following acute MS275 application (Fig. 3A). Gamma band power in the power spectra showed that mean values were not affected by MS275 (n = 6 PPN cells, Mann-Whitney Rank Sum Test, p = 0.9).

We continued by testing the specific HDAC Class II inhibitors tubastatin A (150–500 nM; Class IIb, HDAC 6 inhibitor)^15^, and MC1568 (1 μM; Class IIa inhibitor)^2,16–19^. Acute tubastatin A (150 nM) bath application did not affect gamma oscillations in PPN neurons (n = 5) (Fig. 3B). No change was observed on either membrane potential (n = 5 PPN cells, SB + TTX: −53 ± 2 mV; + tubastatin A 150 nM: −51 ± 2 mV; paired Student’s t-test, df = 4; t = 0.8; p = 0.47), or input resistance of PPN neurons (n = 7 PPN cells, SB + TTX: 175 ± 14 MΩ; + tubastatin A150 nM: 230 ± 42 MΩ; paired Student’s t-test, df = 4; t = 1.88; p = 0.13). Furthermore, tubastatin A (500 nM) did not change the mean power of gamma oscillations in the power spectrum (Paired Student’s t-test, df = 4; t = −0.58; p = 0.59).

**Figure 4:**
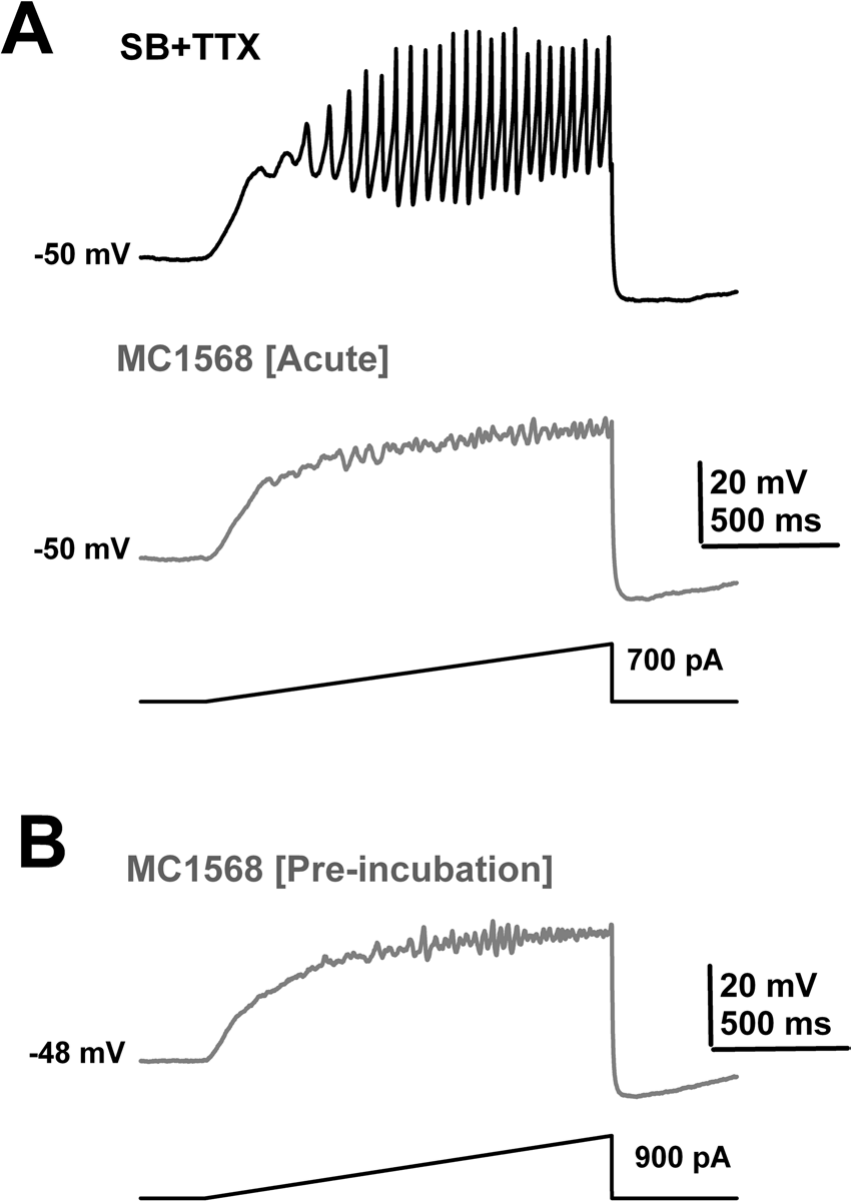
Effects of acute MC1568. (**A**) Ramp-induced oscillations recorded from a PPN neuron before (SB + TTX; black record), and after acute MC1568 exposure (20 min; 1 μM, grey record) showing elimination of fast oscillations and decreased amplitude of oscillations. (**B**) Ramp-induced oscillations in a representative PPN cell showing low amplitude, low frequency oscillations in the presence of SB + TTX after prolonged (pre-incubation) exposure to MC1568 (125 min; 1 μM, pre-incubation).

Acute application of the HDAC Class IIa inhibitor MC1568 did not change the membrane potential (n = 8 PPN cells, SB + TTX: −49 ± 1.0 mV; +MC1568: −50 ± 1.5 mV; paired Student’s t-test, df = 7; t = −0.47; p = 0.65), although it increased the input resistance of PPN neurons by 12 ± 4% (n = 7 PPN cells, SB + TTX: 193 ± 8 MΩ; + MC1568: 217 ± 28 MΩ; paired Student’s t-test, df = 6; t = 2.73; p = 0.034). PPN membrane gamma band oscillations were drastically reduced (Fig. 4A). In addition, pre-incubated PPN slices with MC1568 (1 μM) (90–120 min at 36 °C in the presence of SB + TTX) also reduced the presence of gamma oscillations in these neurons during ramp-induced depolarization (Fig. 4B). The mean reduction of power in the spectra of membrane oscillations before and following acute and pre-incubation with MC1568 in different PPN neurons is shown on Table 3. No statistically significant decreases were observed in the power of lower frequency membrane oscillations after either acute or pre-incubation application of MC1568 (10–15 Hz, 15–20 Hz, or 20–30 Hz; Student’s t-test, p > 0.05) (Table 3). However, under both experimental conditions gamma band (30–100 Hz) power reduction was statistically significant (Table 3).

**Table 3:** Mean power changes (% of control) mediated by MC1568 (1 μM).

	10–15 Hz	15–20 Hz	20–30 Hz	30–100 Hz
Acute effect (n = 8)	62.7 ± 14.9%	80.3 ± 26.6%	69.2 ± 38.4%	37.7 ± 13.5%^**#**^
Pre-incubation effect	108.7 ± 38.4%	59.3 ± 11.4%	48.4 ± 33.8%	2.4 ± 10.9%*
(n = 25)				

^#^p < 0.05, paired Student’s t-test (df = 7, t = 4.311; p = 0.004). *p < 0.05; One-way ANOVA, (F(1,23) = 7.43; Bonferroni’s post-hoc t-test, t = 2.73, p = 0.012).

### Effects of HDAC Class I and II inhibitors on CaMKII modulation

**Figure 5:**
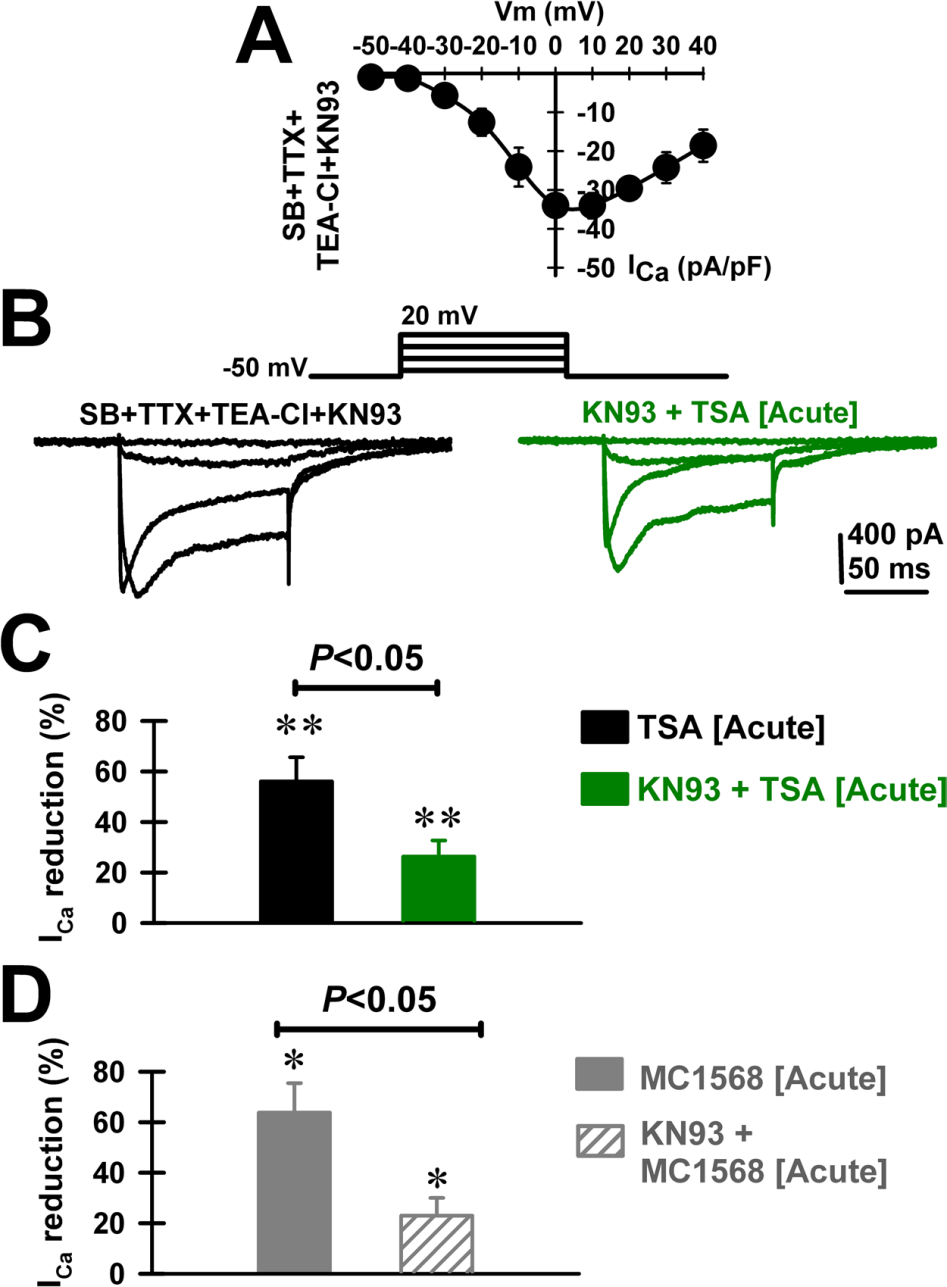
Effects of pre-incubation with the Ca^2+^-calmodulin‐dependent kinase type II (CaMKII) inhibitor KN-93 (1 μM) on the TSA- (1 μM) and MC1568- (1 μM) induced reduction of I_Ca_. (A) Standard current-voltage (I-V) relationship of I_Ca_ after 30 min incubation with KN-93 (n = 5 PPN neurons). (**B**) Representative I_Ca_ recorded using 100 msec long depolarizing square steps from a holding potential of −50 mV to 0 mV after 30 min incubation with KN-93 (1 μM; black record) and after acute bath application TSA (20 min, 1 μM; green record). (**B**) Percent reduction in I_Ca_ amplitude in a group of PPN cells (n = 8) recorded after acute bath application TSA (20 min, 1 μM; black bar), and following pre-incubation with KN-93 (1 μM; n = 7; green bar). **p < 0.01 significant reduction of I_Ca_ by acute bath application of TSA (Paired Student’s t-test; t = −3.77; df = 7; *p* = 0.007), or KN93 + TSA (paired Student’s t-test; t = −3.96; df = 6; *p* = 0.007). Note the statistically significant KN-93-dependent blunting effect of the TSA-induced reduction of I_Ca_ (One-way ANOVA; F_(1,13)_ = 6.3; *p* < 0.05; post-hoc Bonferroni’s *t*-test, *t* = 2.51, *p* = 0.03). (**C**) Percent reduction in I_Ca_ amplitude in a group of PPN cells (n = 6) recorded after acute bath application MC1568 (20 min, 1 μM; solid grey bar), and following pre-incubation with KN-93 (1 μM; n = 6; dashed grey bar). *p < 0.05 significant reduction of I_Ca_ by acute bath application of MC1568 (n = 6 PPN cells; paired Student’s t-test, t = 3.46; df = 5, p = 0.018), or KN93 + MC1568 (n = 6 PPN cells; paired Student’s t-test, t = −3.75; df = 5, p = 0.013). Note the statistically significant blunting effect of KN-93 on the mean I_Ca_ reduction by MC1568 (One-way ANOVA; F_(1, 10)_ = 9.24; p = 0.013; post-hoc Bonferroni *t*-test; t = 3.04; *p* < 0.05).

We further studied whether the TSA- and MC1568-mediated reductions of I_Ca_ required intracellular Ca^2+^-calmodulin‐dependent kinase type II (CaMKII) activation. Pre-incubating PPN slices with KN-93 (1 μM; >30 minutes; in SB + TTX at 36 °C) recorded I_Ca_ showed substantial mean current density and voltage dependence (Fig. 5A). Acute TSA (1 μM) exposure was less effective in reducing I_Ca_ in the presence of KN-93 (Fig. 5B). Indeed, the mean I_Ca_ reduction by TSA decreased from 56.0 ± 9.6% (Fig. 5C, black bar; n = 8; paired Student’s t-test; t = −3.77; df = 7; p = 0.007) to 26.3 ± 6.3% in the presence of KN-93 (Fig. 5C, green bar; n = 7; paired Student’s t-test; t = −3.96; df = 6; p = 0.007). No change on I_Ca_ kinetics was observed during KN-93, KN-93 + TSA, or MC1568 acute application (Table 2). In conclusion, the acute effects of TSA on I_Ca_ were blunted by KN-93 (Fig. 5C; One-way ANOVA; F(1, 13) = 6.3; p < 0.05; Bonferroni’s post-hoc t-test, t = 2.51, p = 0.03). We also observed similar effects of KN-93 on the MC1568-mediated reduction of I_Ca_. The mean reduction of I_Ca_ by MC1568 was blunted by KN-93 (Fig. 5D; One-way ANOVA; F(1, 10) = 9.24; p = 0.013; post-hoc Bonferroni t-test; t = 3.04; p < 0.05). The mean reduction of I_Ca_ by MC1568 decreased from 63.7 ± 11.7% in the absence of KN-93 (n = 6 PPN cells; Student’s t-test, t = 3.46; df = 5, p = 0.018) to 23.0 ± 7.0% (n = 6 PPN cells; Student’s t-test, t = −3.75; df = 5, p = 0.013) after KN-93.

## Discussion

### Gamma oscillations in the PPN

In order to better appreciate the importance of the relationship between HDACs and gamma band oscillations in PPN neurons, we need to understand the role of gamma frequency oscillations in this region. During waking and REM sleep, the EEG manifests low amplitude, high frequency activity in the beta/gamma frequency range (~20–30/30–90 Hz)^20^. The PPN is most active during the two states of high frequency activity, waking and REM sleep^21^. The PPN is the arm of the RAS that modulates ascending projections through the thalamus to modulate arousal, and descending projections through the pons and medulla to modulate posture and locomotion^21^. The PPN is composed of different populations of cholinergic, glutamatergic, and GABAergic neurons^22^. PPN neurons exhibit beta/gamma frequencies *in vivo* during active waking and REM sleep, but not during slow wave sleep^23–28^. Moreover, the presence of gamma band activity has been confirmed in the cortical EEG and PPN of the cat *in vivo* when the animal is active;^24^ and in the region of the PPN in humans during stepping, but not at rest^29^. PPN neurons were found to fire at low frequencies ~10 Hz at rest, but the same neurons increased firing to gamma band frequencies when the animal woke up, or when the animal began walking on a treadmill^30^. That is, the same cells were involved in both arousal and motor control. Thus, there is convincing evidence for the presence of gamma band activity during active waking and movement in the PPN *in vitro*, *in vivo*, and in a number of species.

We described the mechanisms behind gamma band activity in the PPN^9–13,21^, and they will not be reiterated. Briefly, intrinsic gamma oscillations are mediated by voltage-dependent, high threshold N- and P/Q-type Ca^2+^ channels that are present in every PPN neuron, regardless of cell or transmitter type. The two Ca^2+^ channel subtypes are modulated by different intracellular pathways, specifically, N-type and P/Q-type channels are modulated by the cAMP/PK pathway and the CaMKII pathway, respectively. Moreover, there are three cell types in the PPN, those bearing only N-type Ca^2+^ channels (N-only cells), those with both N- and P/Q-type (N + P/Q cells), and those with only P/Q-type Ca^2+^ channels (P/Q-only cells)^9,31–33^. These results suggest that there is a “waking” pathway mediated by CaMKII and P/Q-type channels and a “REM sleep” pathway mediated by cAMP/PKA and N-type channels. These findings also suggest that different PPN cells may fire during waking (those with N + P/Q or “Wake-REM on”, and P/Q-only, or “Wake-on” cells) vs REM sleep (those with N + P/Q, or “Wake-REM on”, and only N-type, or “REM on” cells)^10^.

### New Findings

**Figure 6:**
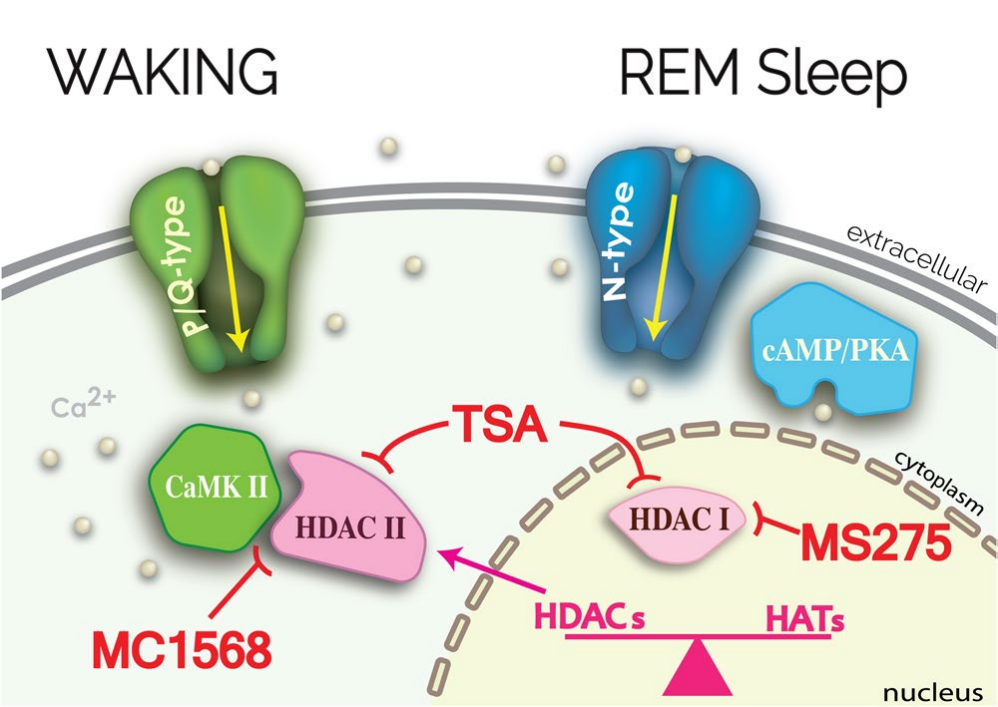
Summary of results. P/Q-type channels are modulated by CaMKII to generate gamma band oscillations (green structures). TSA blocked these oscillations and reduced I_Ca_ in PPN cells by inhibiting HDAC I and II (pink structures). KN-93 blunted the effects of TSA, suggesting that HDAC II action on CaMKII may be required for potential transcription following P/Q-type channel-mediated gamma oscillations. Inhibition of HDAC I by MS275, or of HDAC IIb by tubastatin A, in the nucleus had no effects on I_Ca_ or oscillations. However, MC1568 inhibition of HDAC IIa had the same effect as TSA in reducing I_Ca_ and oscillations. These effects were evident in cells with P/Q-type channels (N + P/Q and P/Q-only cells) but there were no effects on N-only cells that are modulated by cAMP/PKA (blue structures).

The present results showed that, (a) acute *in vitro* exposure to the histone deacetylation Class I and II inhibitor TSA led to the elimination of high threshold, voltage-dependent Ca^2+^ channel-mediated oscillations, specifically in the gamma band range, but not lower frequencies, (b) pre-incubation with TSA led to a similar decrease specifically in gamma band oscillations, (c) a significant reduction in I_Ca_ was elicited by TSA, especially on cells with P/Q-type channels and less so on N-only cells, (d) a HDAC Class I inhibitor MS275, and a Class IIb inhibitor tubastatin A, both failed to affect intrinsic gamma oscillations in PPN neurons, (e) MC1568, a HDAC Class IIa inhibitor, blocked gamma oscillations, and f) the effects of both TSA and MC1568 were blunted by blockade of CaMKII with KN-93. Figure 6 provides a summary of the findings described.

The studies described have certain limitations that should be noted, especially since the results shown are among the first to address histone acetylation/deacetylation in this part of the brain. The PPN is composed of three types of neurons previously described to express different neurotransmitters and have different *dendritic branching*^22^ that would result in different input resistance ranges. Furthermore, input resistance values were consistent with our previous reports^13^. Moreover, TSA has a host of nuclear and cytoplasmic actions affecting a number of mechanisms. Biochemical studies showed a direct inhibitory effect of TSA on HDAC classes I and II^34^. Acute treatment with TSA blunted seizure-triggered down-regulation of glutamate receptor expression in hippocampal CA3 neurons;^35^ and one hour of acute incubation with TSA (1 μM) occluded synaptic activity-mediated nuclear transcription factor activation of hippocampal neurons in culture^36^. Moreover, chronic treatment with TSA showed neuroprotective effects against losing mitochondrial Ca^2+^ homeostasis in striatal neurons in culture^37^. Finally, TSA was initially used to prevent cochlear cell death, followed by a result showing that specific inhibitors that HDAC6 (i.e., a HDAC class II) mediated the TSA effects^38^. Our methods tried to limit those actions by using synaptic blockers to eliminate fast synaptic transmission, as well as TTX to prevent action potential generation and thus circuit effects. The remaining activity, therefore, was mostly that of intrinsic membrane oscillations, while most synaptic/receptor interactions were negated. Moreover, these oscillations were induced only on the recorded cell by using ramp stimuli, limiting the manifestation of the effects of TSA to those oscillations. These effects were not unspecific since TSA produced frequency- and cell type-specific effects, i.e. TSA reduced oscillations in the gamma range but not lower frequency oscillations, and TSA reduced oscillations mainly in cells with P/Q-type channels but not in cells with N-type channels. TSA did not affect low frequency oscillations, having a statistically significant effect only on >30 Hz oscillations. TSA also showed cell specificity since it did not significantly affect N-only PPN neurons, which displayed normal gamma oscillations. Its effects were mainly on N + P/Q and P/Q-only cells.

Another issue is that of timing. Transcription is generally thought to occur in the range of hours to days, therefore, could changes in gene transcription by TSA and MC1568 occur during the time frame studied here? We know that agents like TSA and MC1568 can act within 15–30 min to modulate immediate early genes like *c-fos* and *c-jun*, at least in other cell types^39,40^. Thus, we can assume that at least some transcription is taking place during the recording period. It remains to be determined through genomic and proteomic analyses which genes and proteins may be affected by the manifestation of gamma oscillations. Such analysis is ongoing and beyond the current scope. Such studies are notoriously difficult on the brain because of the cellular heterogeneity and the multitude of receptors and channels involved. However, the methods employed here limited activity to intrinsic membrane oscillations in a nucleus in which every cell manifests gamma oscillations. As such, the functional homogeneity of the PPN makes it an ideal site for genomic and proteomic study.

We are careful not to conclude that any form of gene transcription takes place as a result of the manifestation of gamma band oscillations, or change as a result of HDAC inhibition. Given that proteomic and genomic analyses are not included herein, such conclusions would be premature. The results, however, are of exceptional value in demonstrating that gamma oscillations are linked to processes that may be involved in gene transcription.

### Clinical Implications

Upon waking, sensory information onto PPN neurons induces arousal, representing a continuous flow of afferent input^21^. The role of gamma band activity in PPN neurons has been proposed to stabilize coherence related to arousal, providing a stable state during waking^13,41,42^. This constant process was suggested to help evaluate the world around us on a continuing basis, that is, to bestow preconscious awareness^9–12^. The sustained flux of afferent information was termed “bottom-up gamma”^43,44^, which may provide a remodeling of neurons in this part of the RAS, a daily update of sensory experience. The results described above suggest that the continuous flow of information may modulate at least some processes involved in transcription, which could represent one mechanism behind such remodeling. In addition, the PPN relays gamma band activity to cells in the intralaminar thalamus, which also express N- and P/Q-type Ca^2+^ channels^45,46^. The intralaminar thalamus projects to the cortex to elicit arousal, but its effects on the cortex differ between waking and REM sleep. Coherence in the EEG between distant sites on the cortex is present during waking, but is absent during REM sleep^47,48^. That is, the brainstem appears to dictate cortical EEG activity but does so differently during waking *vs* REM sleep. Our results suggest that the action of TSA was restricted mainly to cells with P/Q-type channels, which are thought to be active during waking but not REM sleep^9–12^. Neuroepigenetic modulation of these PPN neurons through these particular HDAC mechanisms may occur specifically during waking.

Clinically, we see at least two possible new directions of research to test the involvement of this mechanism in certain disorders. First, blocking histone deacetylation with TSA may have deleterious effects on intrinsic gamma oscillations in the PPN, inducing an inhibition of arousal, which accounts for some of the side effects of this medication. Importantly, the modulation of processes related to transcription during waking could have additional consequences. Second, we previously proposed that insomnia represents not too little sleep but too much waking^10,21,43^. We proposed that insomnia, in at least some cases, may be due to over expression of P/Q-type Ca^2+^ channels, since those were proposed to be preferentially active during waking. A potential process by which this could occur is through transcription leading to over expression of P/Q-type channels. Moreover, we know that gene regulation can be mediated by voltage-dependent Ca^2+^ channels^49^, and that a number of human disorders result when P/Q-type channels mutate or become dysfunctional^50^. These findings point to a larger number of potentially fruitful areas of research that need to be pursued.

## Methods

### Slice preparation

Pups aged 9–13 days from adult timed-pregnant Sprague-Dawley rats (280–350 g) were anesthetized with ketamine (70 mg/kg, I.M.) until tail pinch reflex was absent. This age range was selected due to the developmental decrease in REM sleep of the rat that occurs between 10 and 30 days^51^. This period of investigation enabled sampling from a baseline period (9–13 days), as determined by our previous body of work on the PPN^52^. The PPN is the only cell group in the RAS in which every neuron manifests intrinsic gamma oscillations^9,10^. Basically, the rat undergoes a developmental decrease in REM sleep from about 10 to 30 days of life, with the most significant changes occurring after 14–15 days. The time period studied is a stable epoch during which an exuberant, intrinsic gamma oscillations *in vitro* correlates with higher REM sleep *in vivo* during development that then decreases to the adult level by 30 days. Pups were decapitated and the brain was rapidly removed then cooled in oxygenated sucrose-artificial cerebrospinal fluid (sucrose-aCSF). The sucrose-aCSF consisted of (in mM): 233.7 sucrose, 26 NaHCO_3_, 3 KCl, 8 MgCl_2_, 0.5 CaCl_2_, 20 glucose, 0.4 ascorbic acid, and 2 sodium pyruvate. Sagittal sections (400 μm) containing the PPN were cut and slices were allowed to equilibrate in normal aCSF at room temperature. The aCSF was composed of (in mM): 117 NaCl, 4.7 KCl, 1.2 MgCl_2_, 2.5 CaCl_2_, 1.2 NaH_2_PO_4_, 24.9 NaHCO_3_, and 11.5 glucose. Slices were recorded at 36 °C while perfused (1.5 ml/min) with oxygenated (95% O_2_–5% CO_2_) aCSF in an immersion chamber for patch clamp studies as previously described^13,14,45^. During recordings, aCSF solution contained the following synaptic receptor antagonists: the selective NMDA receptor antagonist 2-amino-5-phosphonovaleric acid (APV, 40 μM), the competitive AMPA/kainate glutamate receptor antagonist 6-cyano-7-nitroquinoxaline-2,3-dione (CNQX, 10 μM), the glycine receptor antagonist strychnine (STR, 10 μM), the specific GABAA receptor antagonist gabazine (GBZ, 10 μM), and the nicotinic receptor antagonist mecamylamine (MEC, 10 μM) collectively referred to here as synaptic blockers (SB). We also used the sodium channel blocker tetrodotoxin (TTX, 3 μM). All experimental protocols were approved by the Institutional Animal Care and Use Committee of the University of Arkansas for Medical Sciences and were in agreement with the “Guidelines for the Care and Use of Mammals in Neuroscience and Behavioral Research” (National Research Council, 2003), used by the National Institutes of Health for the care and use of laboratory animals.

### Whole-cell patch-clamp recordings

Differential interference contrast optics was used to visualize neurons using an upright microscope (Nikon FN-1, Nikon, USA). Whole-cell recordings were performed using borosilicate glass capillaries pulled on a P-97 puller (Sutter Instrument Company, Novato, CA) and filled with a high-K^+^ intracellular solution, designed to mimic the intracellular electrolyte concentration, of (in mM): 110 K^+^-Gluconate; 30 KCl; 10 Hepes; 10 Na_2_ phosphocreatine; 0.2 EGTA; 2 Mg-ATP; 0.5 Li-GTP; 1 MgCl_2_. Osmolarity was adjusted to ~270–290 mOsm and pH to 7.3. The pipette resistance was 2–5 MΩ. All recordings were made using a Multiclamp 700B amplifier (Molecular Devices, Sunnyvale, CA) in both current and voltage clamp mode. Digital signals were low-pass filtered at 2 kHz, and digitized at 5 kHz using a Digidata-1440A interface and pClamp10 software (Molecular Devices). We used 1.5 sec-long depolarizing current ramps to study membrane potential oscillations. Current ramps allowed us to gradually change membrane potential from resting values up to 0 mV in current clamp mode^13,14,45^. Input resistance was calculated using square hyperpolarizing current pulses (−300 pA, 500 msec long). When recorded cells expressed HCN channels, membrane resistance was calculated after the membrane was charged and immediately prior to voltage rectification.

The recorded neurons were localized in the *pars compacta* in the posterior PPN, which is easily identified in sagittal sections of the brainstem^13,14,41,45^. The recording region was located mainly in the *pars compacta* in the posterior PPN, immediately dorsal to the superior cerebellar peduncle. This area of PPN has been shown to have the highest density of cells^22,53^. We first identified PPN neurons by cell type as previously described^41,52,54^. Gigaseal formation and further access to the intracellular neuronal compartment were achieved in voltage-clamp configuration mode. Within a short time after rupturing the membrane, the intracellular solution reached equilibrium with the pipette solution without significant changes in either series resistance (ranging 7–15 MΩ) or membrane capacitance values.

Voltage-dependent Ca^2+^ currents (I_Ca_) were studied using a high-Cs+/QX314 pipette solution (in mM: 110 CsMeSO_3_, 40 HEPES, 10 TEA-Cl, 12 Na_2_-phosphocreatine, 0.5 EGTA, 2 Mg-ATP, 0.5 Li-GTP, and 1 MgCl_2_. pH was adjusted to 7.3 with CsOH). Cesium and TEA-Cl are widely used potassium channel blockers. I_Ca_ was recorded in the presence of extracellular synaptic receptor antagonists^13,14,45^, and the sodium and potassium channel blockers tetrodotoxin TTX (3 μM) and TEA-Cl (25 μM), respectively. Square voltage steps (100 msec long) were used to generate PPN neuronal I_Ca_ from a holding potential of −50 mV, and then depolarized up to 40 mV. Setting the holding potential at −50 mV allowed us to inactivate T-type Ca^2+^ channels, while allowing normal activation of high-threshold P/Q- and N-type Ca^2+^ channels that have been described by our group to mediate membrane potential oscillations^13,14,31,45^. Both series resistance and liquid junction potential were compensated (>14 kHz correction bandwidth; equivalent to <10 msec lag). Time course of activation (τ_ON_) and deactivation (τ_OFF_) of calcium currents were obtained after fitting individual currents to the function y = y_0_ + a × exp (−Time (ms)/τ_(ON or OFF))._ τ_ON_ was obtained from baseline to the peak I_Ca_ achieved during initial pulse depolarization, while τ_OFF_ was obtained fitting the deactivation from peak tail currents to baseline^14^. As shown previously, we used the specific N-type channel blocker ω-Conotoxin-GVIA (ω-CgTx, 2.5 μM) to identify PPN cell types according to the Ca^2+^ channels type they expressed^31–33^. If I_Ca_ was blocked completely, the cell was classified as N-only, if it was blocked partially, it was classified as N + P/Q, and if it was not affected, it was classified as P/Q-only.

### Drug application

Bath-applied drugs were administered to the slice via a peristaltic pump (Cole-Parmer, Vernon Hills, IL), and a three-way valve system such that solutions reached the slice 1.5 min after the start of application. Drugs reached a steady state maximal concentration after 10 min. In this study, we observed maximum effects of histone deacetylase inhibitors after 20 min, and lasted for up to 120 min. The sodium channel blocker TTX, as well as tetraethylammonium (TEA-Cl) were purchased from Sigma Aldrich (St. Louis, MO). KN‐93, a selective inhibitor of Ca^2+^-calmodulin‐dependent kinase type II (CaMKII), was purchased from Cayman (caymanchem.com). Trichostatin-A [(2E,4E,6 R)-7-(4-(Dimethylamino)phenyl)-N-hydroxy-4,6-dimethyl-7-oxo-2,4-heptadienamide], MS275 [Pyridin-3-yl)methyl 4-(2-aminophenylcarbamoyl)benzylcarbamate], MC1568 [3-[5-(3-(3-Fluorophenyl)−3-oxopropen-1-yl)-1-methyl-1*H*-pyrrol-2-yl]-*N*-hydroxy-2-propenamide], and tubastatin A hydrochloride [N-Hydroxy-4-[(1,2,3,4-tetrahydro-2-methyl-5H-pyrido[4,3-b]indol-5-yl)methyl]benzamide hydrochloride] were purchased from TOCRIS (Minneapolis, MN). Stock solution of histone deacetylase (HDAC) inhibitors used in this study were dissolved in DMSO and immediately stored at −30 °C. The final concentration of DMSO in the recording aCSF solution was <0.01%. Control aCSF contained the same DMSO concentration during control, SB + TTX, and pre-drug recording conditions. We based our working concentrations of HDAC inhibitors on previously published reports using neurons *in vitro*. Trichostatin A (TSA) at 250 nM and 1 μM concentrations were 1.6 and 6.5 times higher, respectively, than IC_50_ values reported previously in cell cultures^50^, and 10 times higher than the lowest effective concentration used on dopaminergic neurons *in vitro*^55^. The same TSA concentrations were used in hippocampal dissociated neurons in culture^4^ as well as on organotypic slices in culture^5^. MS275 500 nM and MC1568 1 μM concentrations were shown to yield maximal effects after pre-incubation *in vitro* for dopaminergic cell cultures^56,57^. MC1568 at 1 μM corresponded to a 10 times higher concentration than IC_50_ values reported using tumor cell lines in culture^16,17^. Tubastatin A 100 nM and 500 nM concentrations were 10 and 30 times higher, respectively, than IC_50_ values reported previously^15^. That is, based on the differential affinities, the final concentrations used were 1 μM for both TSA (6.5x IC_50_), and MC1568 (10x IC_50_), and 500 nM for MS275 (10x IC_50_) and tubastatin A (30x IC_50_).

### Data Analysis

Off-line analyses were performed using Clampfit software (Molecular Devices, Sunnyvale, CA). Peak oscillatory amplitude was analyzed by first filtering each ramp recording and measuring the three highest amplitude oscillations to derive a mean amplitude induced during each ramp. The mean peak frequency of the same three oscillations was filtered and measured to derive a mean frequency of oscillations during the three highest amplitude oscillations in each ramp. The power of each frequency was also analyzed by composing a power spectrum for the frequencies in the entire ramp (bandpass filtered high pass 10 Hz, low pass 100 Hz), giving a measure of peak power for frequency^31^. Comparisons between groups were carried out using OriginPro 9.1.0 (Originlab.com, MA, USA) (see Tables 1 and 2). We used either Student’s t-test or One-way ANOVA, with Bonferroni post hoc testing for multiple comparisons. F values and degrees of freedom are reported for all linear regression ANOVAs. Differences were considered significant at values of p ≤ 0.05. All results are presented as mean ± SEM.
